# Proximal TGF-β Signaling in Chronic Injury: from a notorious fibrotic factor to a repair ally

**DOI:** 10.1007/s00424-026-03168-x

**Published:** 2026-04-23

**Authors:** Stellor Nlandu Khodo

**Affiliations:** 1 Department of Physiology, Faculty of Medicine and Sciences, University of Zurich, Zurich, Switzerland; 2 National Institute of Health and Medical Research (INSERM), UMR-S1155, Tenon Hospital, Faculty of Medicine,Sorbonne University, Paris, France

## Abstract

Transforming growth factor beta (TGF-β) is a pleiotropic factor that plays a key role in the pathophysiology of chronic kidney disease (CKD). However, targeting TGF-β inhibition failed to mitigate CKD in practice. The proximal tubule (PT), the most vulnerable renal segment, plays a central role in post-injury repair or the drift to CKD. Despite its well-documented profibrotic effects, recent studies revealed benefits of TGF-β signaling in PT response to CKD, raising the question of its therapeutic potential. This review highlights mechanisms by which TGF-β signaling mediates the PT adaptive response to chronic injury and provides perspectives for developing novel CKD therapy.

**Supplementary Information:**

The online version contains supplementary material available at 10.1007/s00424-026-03168-x.

## Introduction

TGF-β is a multifunctional factor that belongs to a superfamily that includes BMPs and Activins. Three mammalian isoforms (TGF-β1, 2 and 3) have been identified, each encoded by a distinct gene. TGF-β1 is the predominant and best-characterized isoform and will be the focus in this review. It functions through two serine/threonine kinase receptors, type II (TbRII) and Type I (TbRI or ALK5) TGF-β receptors. TbRII binds the active ligand, phosphorylates ALK5, which subsequently activates downstream Smad2/3. Active Smad2/3 oligomerize with Smad4 and translocate to the nucleus where their interactions with transcriptional co-factors lead to expression of a myriad of TGF-β target genes. TGF-β also activates Smad-independent pathways, mainly the MAP kinase pathway [1, 2]. TGF-β signaling is involved in multiple physio-and pathological processes by regulating cell differentiation, inflammation and survival, and displays even opposing effects depending on the dose, the microenvironment and the type of targeted cells. It acts as a tumor suppressor by inducing growth arrest and cell death but can promote malignancy by inducing epithelial to mesenchymal transition (EMT). While genetic or systemic/pharmacological inhibition of TGF-β mitigates renal fibrosis in mouse models of chronic kidney disease (CKD), kidney specific Smad 2 knockout exacerbates TGF-β/Smad3 mediated fibrosis [3–5]. Clinical trials targeting TGF-β inhibition in CKD failed to mitigate progression, suggesting that, though TGF-β is the most potent fibrotic factor in CKD, it is not the “bandmaster” in CKD progression which requires a complex cooperating activation of multiple factors. The complexity of TGF-β signaling is also evidenced by the phenotypic variability of *TbRI/II* mutations. Recent studies revealed beneficial effects of having intact epithelial TGF-β signaling in experimental models of CKD; *TbRII* deletion in proximal tubule (PT) cells worsened tubulointerstitial fibrosis (TIF) by promoting apoptosis, metabolic disturbances, and inflammation, partly, due to impaired β-catenin activation [6, 7]. While the literature exhaustively describes the ambivalence of TGF-β signaling in CKD, this review highlights its beneficial aspects in PT response to chronic injury.

## TGF-β signaling in the proximal tubule: a guardian of its terminal differentiation?

**Fig. 1:**
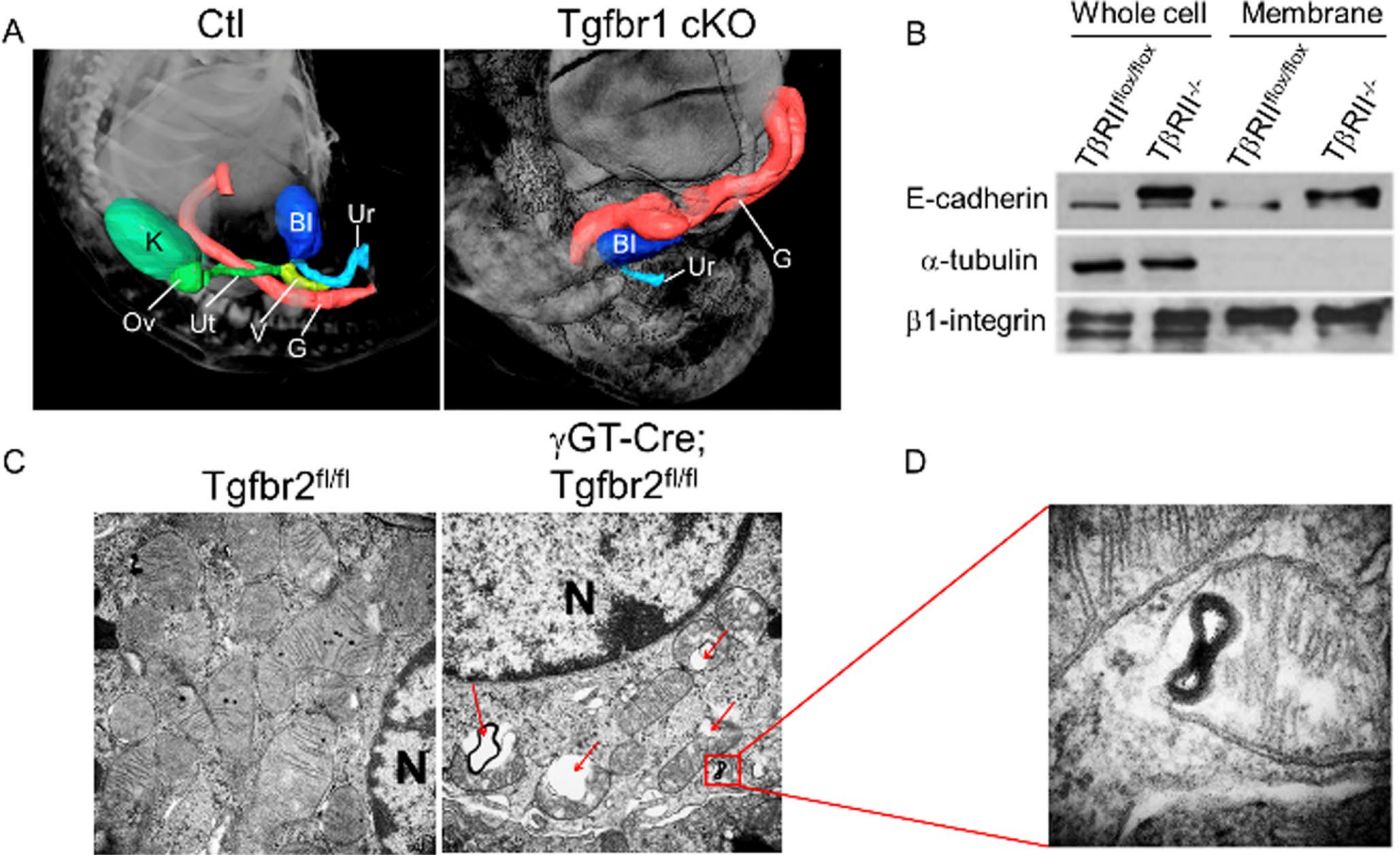
TGF-β signaling in PT terminal differentiation. (**A**) Optical projection tomography 3D reconstruction of organs showing absence of kidneys in Tgfbr1-cKO fetuses. K kidney, G gut; Bl bladder, Ut uterus, V vagina, Ur urethra. (**B**) Immunoblots showing increased E-cadherin in PT cells lacking *TbRII*. (**C**, **D**) Electron microscopy images showing increased PT mitochondrial injury (red arrows) and features of impaired lipid metabolism and mitophagy (accumulation of myelin figures in mitochondrial matrices) in mice lacking Tgfbr2 in their PT (gGT-Cre; Tgfbr2^fl/fl^)

High mitochondrial content and expression of the mesenchymal cadherin, N-cadherin, are two features of fully differentiated proximal tubule (PT) cells, the latter remembering their metanephric mesenchyme (MM) origin. During nephrogenesis, mutual interactions of the MM with the ureteric bud (UB) give rise to the tubular system. Cells from the MM (Six2+) undergo mesenchymal to epithelial transition to form nephron segments except the collecting duct, which emerges from the branching morphogenesis of UB (Wnt11+). In contrast to the rest of the renal epithelia expressing barrier claudins (claudin-1, 4, 7, and others) and epithelial cadherin (E-cadherin), PT cells form a loose and leaky epithelium by expressing pore claudins (claudin-2, 10b) and N-cadherin. TGF-β signaling is required for kidney morphogenesis, partly by modulating HGF-induced branching morphogenesis and promoting tubule elongation [8, 9]. Some studies reported a positive regulatory effect of TGF-β signaling on Six2 expression via Smad3 in mK3 cells. Recently, Lozovska et al. reported that *TbRI* inactivation in mouse caudal embryonic tissue leads to renal agenesis at E16.5, partly due to impaired Wnt/β-catenin activation, implying a pivotal role of this axis in kidney development and post-injury cell plasticity [10] (Fig. 1A). TGF-β signaling promotes N-cadherin expression and is notorious for inducing E-to N-cadherin, an important feature of EMT in CKD and cancers. Abrogation of TGF-β signaling in PT cells switches their mesenchymal N-cadherin to E-cadherin expression (Fig. 1B), suggesting a key role of TGF-β signaling in PT cell terminal differentiation. The PT is responsible for 70% sodium reabsorption which is linearly related to oxygen consumption by mitochondria for ATP production. The structural and functional integrity of mitochondria is determinant for PT cell function and responsiveness to injury. Upon injury, PT cells sense self-and substratum-integrity, secrete growth factors/cytokines to communicate with interstitial cells and facilitate recovery. Unfortunately, this tubulo-interstitial crosstalk usually becomes maladaptive under chronic injury leading to fibrosis. PT cells lacking *TbRII* exhibit increased mitochondrial injury (Fig. 1C, D) and anaerobic glycolysis, mimicking the metabolic feature of medullary renal epithelia. The answer to why PT cells have conserved expression of a mesenchymal cadherin, mostly expressed in high oxygen-dependent cells such as cardiomyocytes and neurons, is likely TGF-β signaling safeguards PT cell terminal differentiation, and that TbRII/N-cadherin interplay is involved in both cell autonomous and epithelio-interstitial communication mechanisms in normal physiology and response to chronic stress.

### Epithelial TGF-β/β-catenin crosstalk in PT adaptive response to chronic injury

**Fig. 2:**
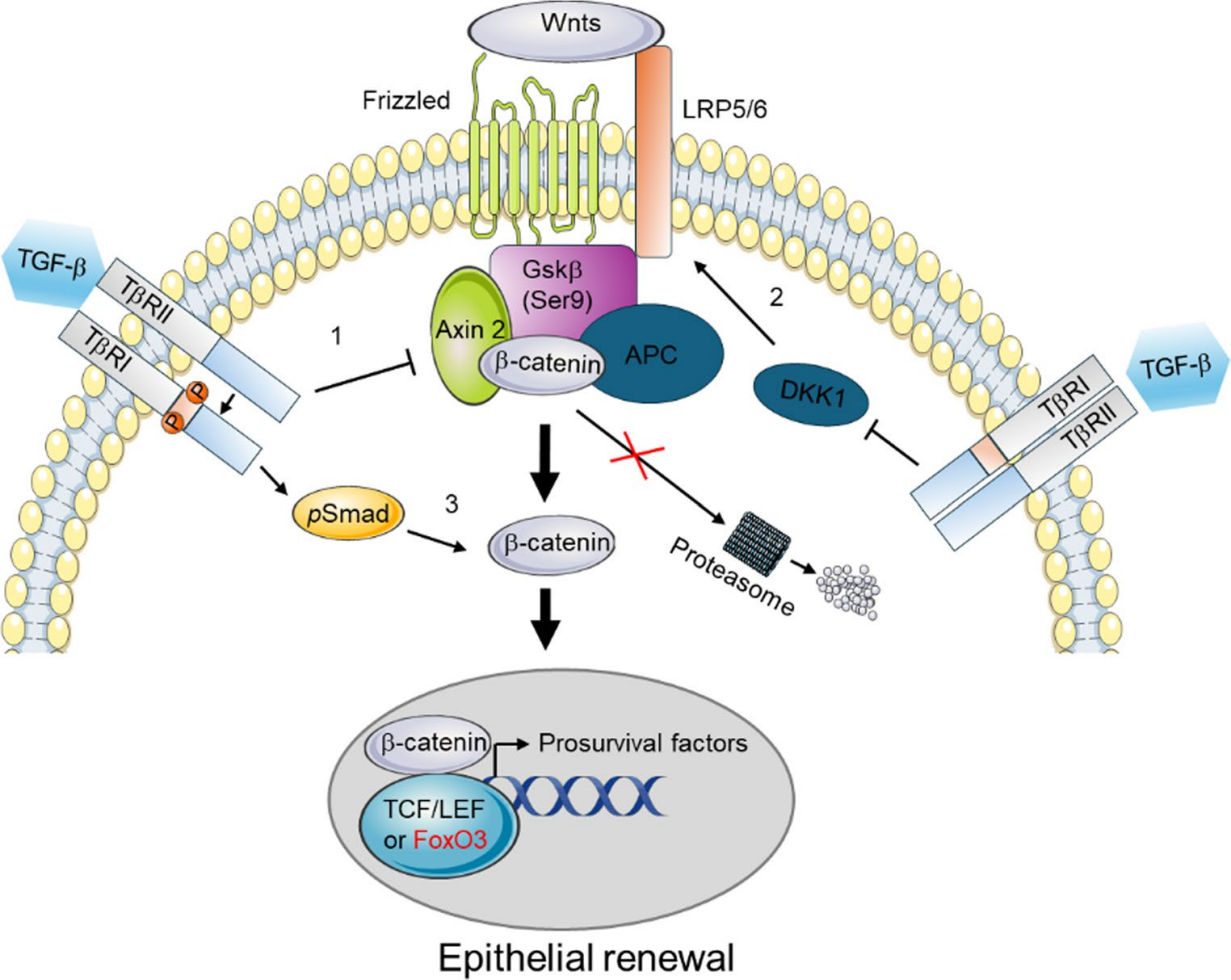
Proximal tubular TGF-β and Wnt/β-catenin crosstalk. Illustration showing how TGF-β receptor signaling impacts Wnt/β-catenin signaling to facilitate self-renewal in homeostasis (β-catenin/TCF/LEF) and upon chronic injury (β-catenin/FoxO3)

Sustained activity of growth factors and cytokines constitutes a self-enhancing loop that promotes organ fibrosis and cancers. β-catenin is a component of cell adherens junctions and the signaling molecule of the Wnt pathway. Mesenchymal TGF-β and Wnt/β-catenin signaling promote fibrosis and CKD progression. However, genetic inhibition of TGF-β signaling in the PT worsens fibrosis, and stabilization of β-catenin in the PT mitigates fibrosis under chronic injury, implying a role of synergistic TGF-β/β-catenin axis in PT adaptive response to chronic injury [6]. Canonical β-catenin activity, which requires its nuclear interactions with the TCF/LEF co-factors, is basally active in the relatively hypoxic renal papilla and transiently enhanced in the renal cortex upon acute kidney injury (AKI). Upon chronic injury, β-catenin favors FoxO3 interaction to mediate cyto-protection and self-renewal. TGF-β/β-catenin axis mediates cell differentiation, which is a prerequisite in post-injury survival prior to proliferation. According to the literature, TGF-β may regulate β-catenin activation at different levels: (1) prevention of β-catenin degradation through Ser9 phosphorylation of GSK3β and repression of Axin 2, both components of β-catenin destruction complex; (2) inhibition of DKK1, an inhibitor of Wnt signaling LRP5/FZD receptor; and (3) promotion of β-catenin nuclear translocation via direct interaction of *p*SMAD3/β-catenin (Fig. 2). Moreover, TGF-β regulates β-catenin activation by targeting E-cadherin repression. TGF-β’s repressive effect on E-cadherin involves activation of zinc-finger transcription factors, including Snail1, Slug and Twist1. Recent studies in human PT cells (HK-2) reported that histone deacetylases (Hdac), particularly Hdac8, play an important role in regulating the TGF-β repressive effect on E-cadherin expression. Moreover, TGF-β may post-transcriptionally regulate cadherin expression by inducing proteinases, including Sheddases (ADAM 10 and TACE) and other metalloproteases [11]. In adherens junctions, β-catenin physically interacts with the intracellular domains of cadherins with a higher affinity for E-cadherin than N-cadherin. By promoting putative N-cadherin expression in PT cells, TGF-β signaling increases the cytosolic pool of β-catenin and favors its nuclear translocation in PT cells. While TGF-β may have a stemness effect on MM cells by inducing Six2 expression in vitro, Wnt9b-induced β-catenin activation lowers Six2 in undifferentiated progenitors (Cited1+), leading to nephrogenesis commitment. Despite its protective effect on mouse models of CKD, renal stabilization of β-catenin induces primitive neoplasms in the long term and mimics Wilms’ tumor in synergy with K-RAS mutations. Given the complexity of this crosstalk, future studies must molecularly define limits between regeneration, carcinogenesis, and fibrogenesis.

## TGF-β signaling in PT mitochondria and inflammatory response

Mitochondria are the cornerstone of PT function and survival, and their dysfunction has been implicated in a broad range of inherited and acquired renal diseases, including tubular defects (Fanconi and Bartter- like syndromes), cystic disease, AKI, glomerular diseases (FSGS), and CKD. The mechanisms whereby TGF-β signaling modulates mitochondrial homeostasis remain unclear. Studies have shown opposing effects of TGF-β signaling on mitochondrial oxidative phosphorylation (OXPHOS) and ATP production in a dose-dependent manner. While higher doses of TGF-β tend to disrupt mitochondrial function, lower doses ameliorate oxygen consumption rate (OCR). PT cells lacking *TbRII* exhibit decreased basal OCR and increased lactate production, consistent with decreased mitochondrial coupling efficiency and reduced ATP production via OXPHOS. TGF-β signaling likely impacts mitochondrial homeostasis by regulating the expression of DNA polymerase gamma (Polg), the only involved in mtDNA replication, as PT cells lacking *TbRII* exhibit decreased Polg and mtDNA copy number. Moreover, the ubiquinone metabolism and complex I function are the most affected pathways in PT cells lacking the *TbRII*, implying that deletion of *TbRII* induces mitochondrial dysfunction and metabolic switch, partly through impaired expression and function of complex I mt-and nuclear DNA encoded subunits. Mitochondrial homeostasis involves quality control, which is mainly mediated by their renewal through biogenesis and mitophagy. PT cells lacking *TbRII* show decreased Pink1 protein, an important regulator of mitophagy, suggesting that TGF-β signaling regulates mitochondrial homeostasis, partly by promoting adequate lipid metabolism and mitophagy (Fig. 1C, D).

TGF-β displays both anti-and pro-inflammatory responses depending on the microenvironment and cell types. However, global *TbRII* or *TGF-b* knockout causes lethal inflammatory disorders in mice, notably by regulating lymphocyte homeostasis [12, 13], implying its intrinsic anti-inflammatory function. Excessive TGF-β signaling in CKD enhances renal inflammation, partly through NF-kB activity. Damage-associated molecular patterns (DAMPS) in CKD bridge mitochondrial injury and inflammation by activating the Cgas/Sting/IFNg axis [14]. Though macrophages and dendritic cells initiate inflammation in response to tubular injury, T cells and macrophages are later involved in the whole evolution of CKD and fibrogenesis. TGF-β signaling promotes pro-repair M2 macrophage phenotype, and inhibiting TGF-β signaling in the PT increases IFNg and TNFα+ CD4 + cells in kidneys under aristolochic acid (AA)-induced CKD. Moreover, the reno-protective Foxp3+ (Treg) CD4 + cells among CD45 + cells are decreased in mice lacking *TbRII* in the PT cells [7]. Taken together, the mitochondrial homeostatic effect of TGF-β signaling in PT seemingly constitutes a guardrail of its anti-inflammatory effect upon chronic injury.

## TGF-β signaling in post-injury cell cycle arrest: a matter of dose?

Cell cycle arrest dysfunction contributes to the pathogenesis of chronic kidney disease (CKD). Renal epithelia are mostly quiescent in G0 with limited cell cycling. Upon injury, depending on severity and frequency, entry into the cell cycle may lead to endocycling-induced polyploidy, G2/M arrest, and an acquired profibrotic Senescent-Associated Secretory Phenotype (SASP), a determinant of the AKI-to-CKD transition. The G1 phase of the cell cycle is an intermediate, stable state during which two daughter cells exiting mitosis sense and integrate their microenvironmental stimuli and decide to enter a resting stage (G0), commit to a new cycle, or die. Cell cycle integrity is tightly regulated by the coordinated interactions of Cyclins and Cyclin-dependent kinases (CDKs), which promote G1 progression to S. TGF-β signaling regulates both G2/M and G1 cell cycle arrest in a dose-and context-dependent manners. High dose (10-20ng/ml) of TGF-β promotes G2/M arrest while lower dose (0.5-1 ng/ml) promotes G0/G1 arrest. G0/G1 arrest is likely the mechanism by which TGF-β promote survival in CKD by inhibiting progression into G2. Studies have demonstrated that epithelial cells in either G0 or G1 have improved survival in response to chronic stress (oxidative stress, hypoxia, toxins, …) than cells at later stages (S, G2/M), which are more vulnerable to apoptosis. TGF-β stimulates G1 arrest in epithelial cells through multiple pathways that target inhibition of CDK/Cyclins, notably Cyclin-dependent kinase inhibitors (CKIs). Two families of CKIs that play an important role in regulating TGF-β-dependent G1 arrest are the INK4 and Cip/Kip family [15]. An FDA-approved CDK4/6 inhibitor, Palbociclib, protected renal function in a murine model of acute kidney injury and CKD [16]. TGF-β signaling in renal epithelia increases transcription of INK4 proteins (e.g. p15INK4B) but not of Cip/Kip family members p21 or p27. Some downstream effectors of TGF-β signaling, such as FoxO1 transcription factor, are known to induce G1 arrest and mediate cytoprotection against oxidative stress by targeting similar CDK inhibitors. FoxO has been reported to interact with Smads and promote transcription of the p15INK4B gene, a well-described target of TGF-β. TGF-β signaling likely augments FoxO1-mediated G1 arrest by increasing FoxO1 transcription and by promoting interactions with Smads to enhance CKI expression [17].

**Fig. 3:**
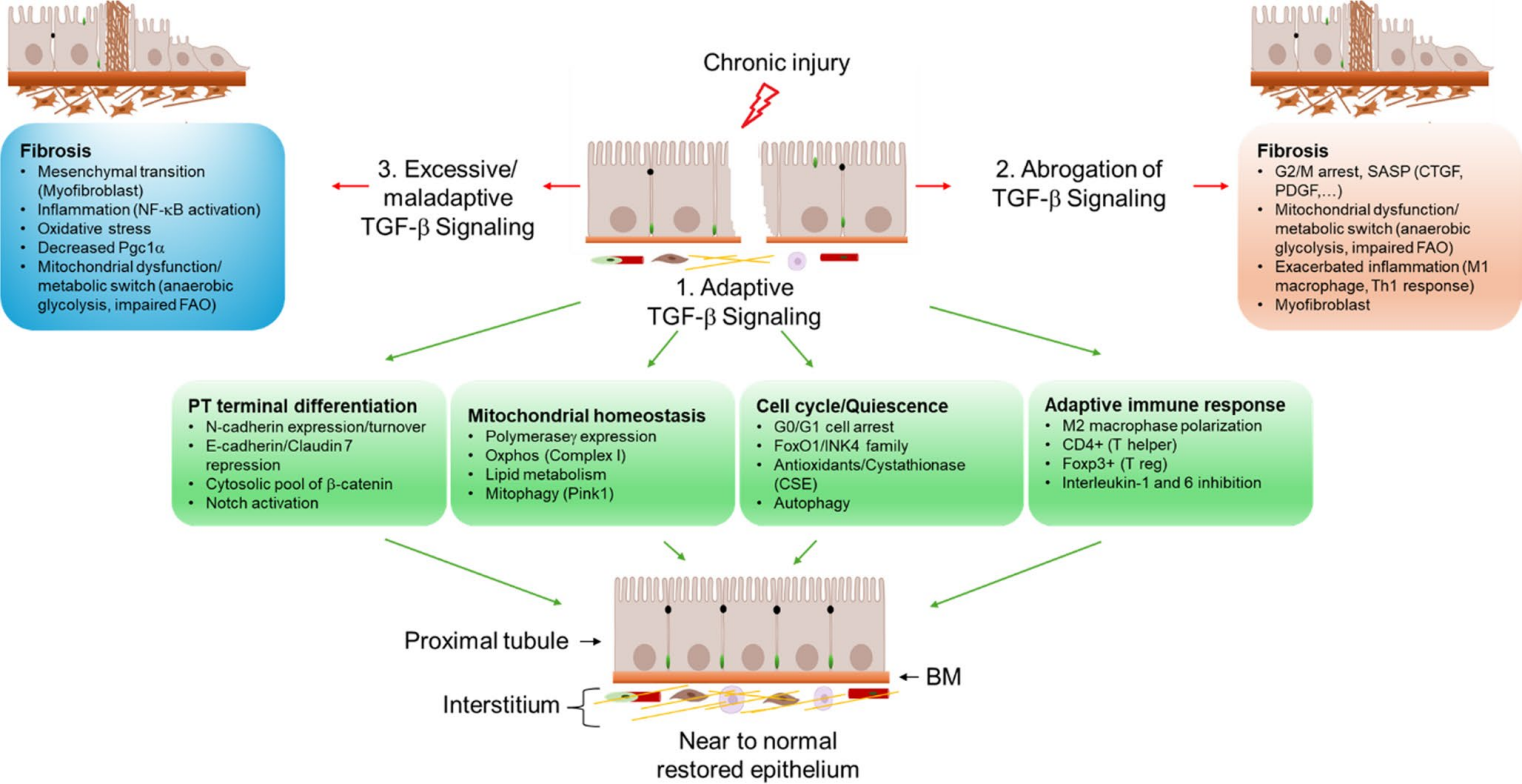
Ambivalent effects of proximal TGF-β signaling upon chronic injury. (1) TGF-β signaling promotes epithelial repair by maintaining PT cell terminal differentiation, mitochondrial homeostasis, cell quiescence and adaptive immune response. (2) While abrogation of TGF-β signaling mostly induces fibrogenesis though cell autonomous mechanisms leading to acquired profibrotic secretory phenotype, mitochondrial dysfunction exacerbated inflammatory response, (3) excessive TGF-β signaling primarily exacerbates myofibroblast dedifferentiation of interstitial cells (fibroblasts, pericytes, endothelial cells and resident immune cells) and aberrant immune response including NF-kB pathway activity (3)

## Targeting TGF-β signaling in CKD: Unmet clinical expectations and perspectives

Regardless of the etiology, tubulointerstitial fibrosis (TIF) is the hallmark of CKD progression. Systemic inhibition of TGF-β signaling mitigated TIF in experimental models of CKD; however, specific abrogation of TGF-β signaling in renal epithelia and matrix-producing interstitial cells (MPICs) failed to impede TIF progression [18, 19]. Moreover, Voelker J et al. demonstrated that the addition of a TGF-β1 monoclonal antibody to renin-angiotensin system (RAS) inhibitors did not slow diabetic nephropathy progression, suggesting that TGF-β signaling is not the cornerstone of CKD progression [20]. Therapies for CKD are adjusted based on etiology and disease stage, including diet adjustment, hypertension management, hyperglycemia, and anemia (RAS blockers, ACE inhibitors and Diuretics, SGLT2 inhibitors and GLP-1 receptor agonists, ESAs and PHD inhibitors, and others), and later dialysis or transplantation. Given the kidney’s intrinsic regenerative capacity, promising strategies should shift from fighting aberrant interstitial cell responses to injury to promoting epithelial self-renewal to impede fibrogenesis. This strategy raises questions including, (1) the cellular and molecular contributors in post-injury regeneration and (2) the line between regeneration and carcinogenesis. Although activating TGF-β in renal epithelia is not a plausible option, understanding its ambivalence may unravel the dynamics of expression between maladaptive and adaptive factors in the pathophysiology of CKD and pave the way for self-renewal-based CKD therapy.

Clinical studies have targeted some downstream adaptive mechanisms of TGF-β signaling, notably promotion of mitochondrial homeostasis via mt-antioxidants (SS-31, MitoQ, SOD mimetics, and others) and adaptive immune response (IL-1β and IL-6 inhibitors) (Fig. 3); however, the results have not yet translated into effective therapies in practice. Targeting G0/G1 cell arrest with Palbociclib or similar agents may also be explored for CKD therapy. Finally, as TGF-β/β-catenin crosstalk is proven crucial for kidney development, genetic manipulation of this axis may reveal key cellular and molecular components in post-injury regeneration, how they spatiotemporally integrate this process, and what leads to the drift toward CKD. Moreover, with new spatial omics techniques, cross-species comparative analyses with lower vertebrates that possess innate TGF-β-mediated post-injury renal regeneration should be considered to identify the missing or unwanted link underlying the restricted post-injury regenerative capacity of mammals.

## Conclusion

While most therapeutic strategies focused on modulating interstitial cell activation and failed to mitigate CKD, shifting the focus to epithelial self-renewal may open new avenues for developing novel CKD therapies. The TGF-β/β-catenin axis is likely useful for identifying molecular and cellular contributors to epithelial self-renewal and adaptive interstitial cell activation upon chronic injury and aging. Identification of these contributors is a prerequisite for advancing regenerative medicine in kidney research and may pay the way for novel prophylactic and therapeutic strategies for CKD and kidney transplant patients.

## Supplementary Information

Below is the link to the electronic supplementary material.

Supplementary Material 1

## Data Availability

No datasets were generated or analysed during the current study.
