# Supplementary material for: Proximal TGF-β Signaling in Chronic Injury: from a notorious fibrotic factor to a repair ally
Source: Pflugers Arch. 2026 Apr 23;478(5):40. doi: 10.1007/s00424-026-03168-x (PMC13102887; doi:10.1007/s00424-026-03168-x)
Supplement: Supplementary file 1 — Supplementary Material 1 [file 424_2026_3168_MOESM1_ESM.docx]

**Abbreviations**

**TGF-β**: Transforming growth factor-beta; **TbRI/II**: Type I and II TGF-beta receptors; **BMP**: Bone morphogenic protein; **ALK5**: Activin receptor-like kinase 5; **SMAD**: Suppressor of mothers against decapentaplegic; **MAPK**: Mitogen-activated protein kinases; **SIX2**: SIX homebox2; **CITED1**: Cbp/p300-interacting transactivator 1; **WNT**: Wingless-type MMTV integration site family; Axin2: Axis inhibition protein 2; **APC**: Adenomatous polyposis coli; **FZD**: Frizzled receptor family; **LRP**: Low density lipoprotein receptor-related protein; **DKK**: Dickkopf homolog; **GSK-3b**: Glycogen synthase kinase-3b; **TCF/LEF**: T-cell factor / lymphoid enhancer factor (transcription factor); **NF-kB**: Nuclear factor-kappa B; **FoxO1/3**: Forkhead box O1/3; **K-RAS**: Kristen Rat Sarcoma Viral oncogene homolog; **ATP**: Adenosine Triphosphate; **DNA**: Deoxyribonucleic acid; mtDNA: Mitochondrial deoxyribonucleic acid; **CGAS**: Cyclic GMP-AMP synthase; **STING**: Stimulator of interferon gene**; TNF-α**: Tumor necrosis factor alpha; **CD4/45**: Cluster of differentiation 4/45; **Foxp3**: Forkhead box p3; Treg: Regulatory T cells; **CDK**: Cyclin-dependent kinases; **CKI**: Cyclin-dependent kinase inhibitor; INK4: Inhibitor of CDK4; **CIP/KIP**: CDK interacting protein/Kinase inhibitory protein; **P21**: Cyclin-dependent kinase inhibitory protein-1A; **P27**: Cyclin-dependent kinase inhibitory protein-1B; RAS: Renin-angiotensin system; **ACE**: Angiotensin converting enzyme; **SGLT2**: Sodium-glucose co-transporter 2; **GLP-1**: Glucagon-like peptide-1; **ESAs**: Erythropoiesis-stimulating agents; **PHD**: Prolyl hydroxylase domain enzyme; **AKI**: Acute kidney injury; **CKD**: Chronic kidney disease; **FSGS**: Focal segmental glomerulosclerosis;
